# Association between systemic lupus erythematosus and osteoporosis: a mendelian randomization analysis

**DOI:** 10.1186/s41927-024-00388-5

**Published:** 2024-05-06

**Authors:** Danfeng Xu, Bing Wu

**Affiliations:** 1 Department of Orthopaedics, Shaoxing Central Hospital, Hua-yu Road 1, Keqiao, Shaoxing, 312030 People’s Republic of China; 2 The Central Hospital of Shaoxing University, Shaoxing, China; 3 Central laboratory, Shaoxing Central Hospital, the Central Hospital of Shaoxing University, Shaoxing, China

**Keywords:** Mendelian randomization, Systemic lupus erythematosus, Osteoporosis, Causal relationship

## Abstract

**Background:**

Systemic Lupus Erythematosus (SLE) and Osteoporosis are two prevalent medical conditions. Previous studies have suggested a possible correlation between SLE and osteoporosis, though the underpinning causal relationship remains largely unknown. The current study aimed to elucidate the causal association between SLE and osteoporosis by employing a Mendelian randomization (MR) approach.

**Methods:**

We performed two-sample MR analysis using the inverse variance-weighted (IVW), weighted median, and MR-Egger methods on publicly available summary statistics datasets using a SLE genome-wide association study (GWAS) as an exposure and osteoporosis GWASs in people with East Asia ancestry as outcomes. The pleiotropy and heterogeneity were examined using a variety of techniques, including the MR-Egger intercept, the MR-PRESSO approach, and the Cochran’s Q test.

**Results:**

We selected 26 single-nucleotide polymorphisms from a SLE GWAS as instrumental variables for osteoporosis. The IVW (*p* < 0.05) method results support a potential association between SLE and osteoporosis. MR-Egger intercept (*p* = 0.82) and MR-PRESSO global test (*p* = 0.80) did not suggest evidence of horizontal or directional pleiotropy. Cochran’s Q test (*p* = 0.78) showed that there was no heterogeneity between IVs.

**Conclusion:**

The results of MR analysis indicated that SLE is likely associated with an increased risk of osteoporosis incidence. Our findings highlight the need for increased awareness the potential risk of osteoporosis among SLE patients.

**Supplementary Information:**

The online version contains supplementary material available at 10.1186/s41927-024-00388-5.

## Introduction

Systemic lupus erythematosus (SLE) is a complex autoimmune disorder characterized by the production of autoantibodies and multi-organ involvement [1]. Patients with SLE present with varying clinical manifestations, including musculoskeletal, renal, cardiovascular, and neuropsychiatric symptoms. The prevalence of SLE varies globally, with higher rates reported in African Americans, Hispanics, and Asians than in Caucasians [2–4]. Over the years, the management of SLE has improved significantly, increasing the life expectancy of affected individuals [5, 6]. However, SLE patients still face numerous challenges, such as the increased risk of developing secondary complications like osteoporosis.

Osteoporosis, a skeletal disorder characterized by compromised bone strength and an increased risk of fractures, is a major public health concern worldwide [7]. The association between SLE and osteoporosis has been studied extensively and there is evidence suggesting an increased risk of osteoporosis in patients with SLE [8, 9]. Recently, Orsolini et al. [10]. reported that osteoporosis and fractures are frequently found in patients with SLE and Lai et al. [11]. showed that patients with SLE had a higher incidence of osteoporosis. Possible factors contributing to low bone mineral density in SLE patients include chronic inflammation, glucocorticoid therapy, and the presence of various cytokines produced by immune cells that interfere with bone homeostasis. Lifestyle factors and low vitamin D levels in SLE patients may further contribute to the risk of osteoporosis [12, 13]. Nevertheless, the causal relationship between these two conditions remains largely unknown, and understanding this connection is crucial for effective prevention and management strategies.

Mendelian randomization (MR) is a sophisticated statistical technique that exploits genetic variants as instrumental variables (IVs) to make causal inferences from observational data [14]. By using genetic instruments, MR analysis reduces the potential for confounding and reverse causation, making it a valuable approach for assessing causal relationships between medical conditions and risk factors [15].

Considering the complex interplay between SLE and osteoporosis and the numerous modifiable risk factors involved, assessing the causal relationship between these two medical conditions is vital for better understanding and management of patients. This study examined this puzzle by conducting an MR analysis to investigate the causal relationship between SLE and osteoporosis.

## Methods

### Study design

**Fig. 1:**
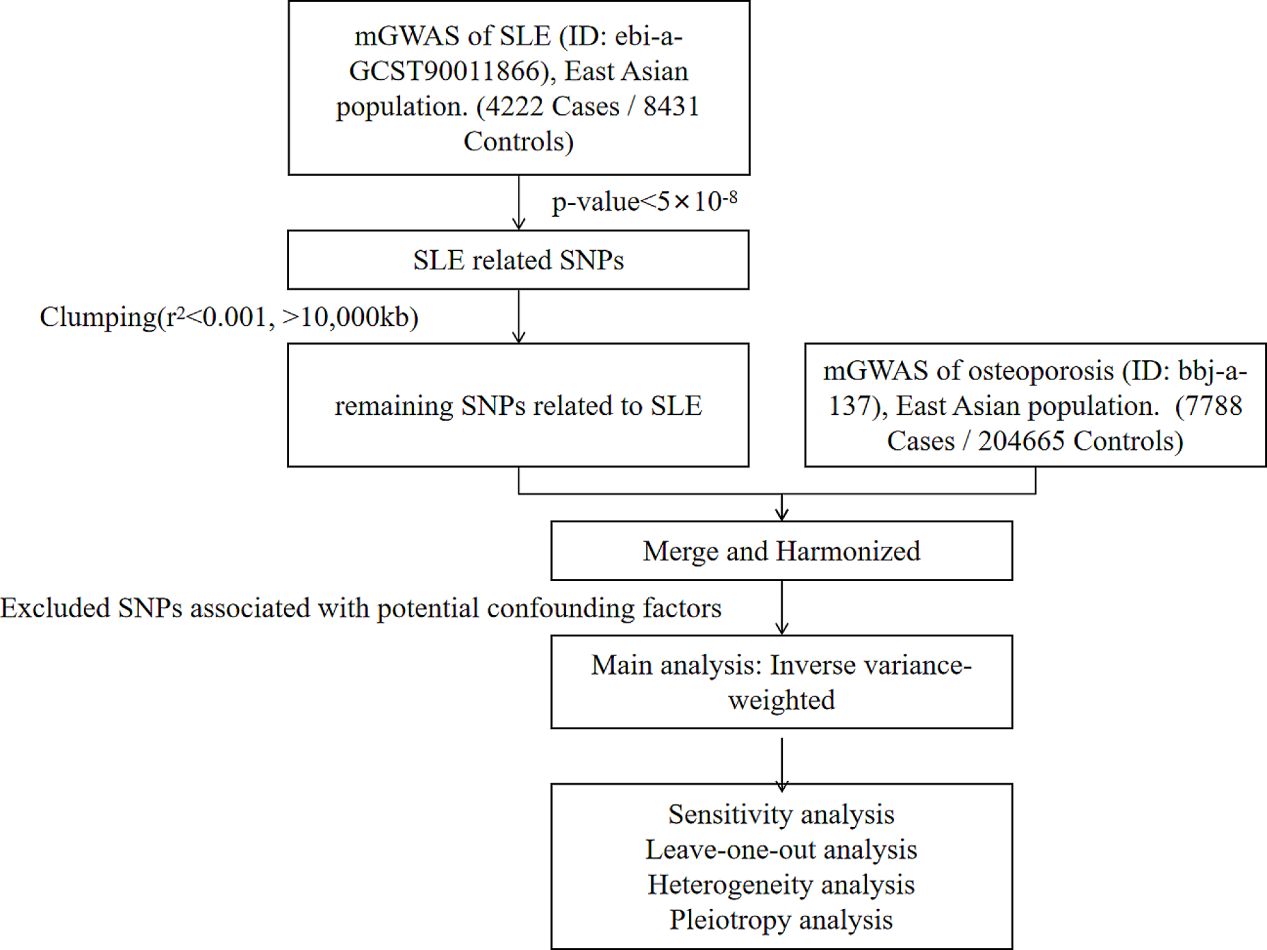
Flowchart of our Mendelian randomization analysis. SLE, Systemic lupus erythematosus; SNPs, single nuclear polymorphisms; GWAS, genome-wide association studies; MR, Mendelian randomization

A two-sample MR study design was used to investigate the causal relationship between SLE as the exposure and osteoporosis as the outcome. This research design is based on the IV method, which uses genetic variants as proxies for the exposure of interest to make unbiased inferences about the causal effects on the outcome. The flowchart was presented in Fig. 1.

### Data sources and genetic instruments

We obtained summary-level genome-wide association study (GWAS) data for SLE and Osteoporosis from publicly available sources. In choosing the exposure and outcome GWAS datasets, we comprehensively considered factors such as population origin, larger sample sizes, more extensive single-nucleotide polymorphism (SNP) coverage, balanced gender composition, and data publication date. To minimize bias due to overlap, we obtained exposure and outcome samples from different databases. For SLE, we used the largest available GWAS, which included 12,653 samples and 5,691,661 SNPs from East Asian (https://gwas.mrcieu.ac.uk/), with the ID number of “ebi-a-GCST90011866” [16]. The GWAS data included 4,222 cases and 8,431 controls. For Osteoporosis, we used GWAS data with 212,453 samples and 8,885,805 SNPs from East Asian (https://gwas.mrcieu.ac.uk/), with the ID number of “bbj-a-137” [17]. The GWAS data included 7,788 cases (696 males and 7,092 females) and 204,665 controls (108,651 males and 96,014 females). Details of the GWAS datasets were provided in supplementary Table 1.

The genetic instruments (or SNPs) were selected based on their strong association with SLE and osteoporosis at a genome-wide significance threshold (*p*-value < 5 × 10^− 8^). Only independent SNPs were retained, after accounting for linkage disequilibrium (r^2^ < 0.001) and clumping (windows size = 10,000 kb) to minimize potential bias due to pleiotropy. The F statistic is calculated as F = beta^2^/se^2^ [18]. The F statistic of IV should be greater than 10. In addition, we remove the SNP directly associated with the outcome (such as bone mineral density, vitamin D, calcium, et al.) by searching on the PhenoScanner website (https://www.phenoscanner.medschl.cam.ac.uk/) [19].

### Data harmonization

The selected SNP data were harmonized between the SLE and osteoporosis datasets through extraction and alignment of effect estimates and standard errors. SNPs were checked for strand alignment, and any palindromic SNPs with intermediate allele frequencies were excluded to avoid any potential ambiguity.

### Mendelian randomization analysis

We used two-sample MR analysis to assess the causal effect of SLE on osteoporosis using the selected SNPs as IVs. For an MR study to provide accurate and reliable estimates, three key assumptions need to be satisfied:

1. Relevance Assumption: The genetic variants used as instruments (SNPs) should be strongly associated with the exposure of interest.
2. Independence Assumption: The genetic variants should be independent of any confounders - variables that are related to both the exposure and the outcome.
3. Exclusion-Restriction Assumption: The genetic variants only affect the outcome through their influence on the exposure, and not through other pathways. This is also known as the absence of pleiotropic effects where the genetic variants (SNPs) cannot directly affect the outcome and do not share any causal pathways with the outcome [20].

We examined different MR methods, including inverse-variance weighted (IVW), weighted median, and MR Egger, each catering to different assumptions and methodological strengths [21]. The IVW method assumes all genetic variants are valid instruments and provide the average causal effect. The MR Egger approach is more robust to pleiotropy but has lower statistical power, while the weighted median method provides consistent estimates even if up to 50% of the SNPs are invalid instruments [22]. Since the precision of the IVW method dependent on the selection of valid SNPs, it could be considered the main tool in the analysis [23, 24].

Heterogeneity among the genetic instruments was assessed using Cochran’s Q statistic with the corresponding *p*-value, and potential pleiotropy was evaluated with the MR-Egger intercept test [25, 26]. The presence of outliers or influential SNPs was appraised through leave-one-out analyses. All analyses were performed using the TwoSampleMR (version 0.5.6) package in R (version 4.3) [27]. *P* < 0.05 was considered indicative of statistical significance.

### Ethical considerations

As this study used publicly available summary-level genome-wide association study data, no additional ethics approval was required.

## Results

After removing the IVs with linkage disequilibrium (31 SNPs remaining) and the SNP directly associated with osteoporosis (5 SNPs excluded), 26 SNPs were obtained in this study. The F-statistic for the SNP exceeds 10. Details were showed in supplementary Table 2.

**Fig. 2:**
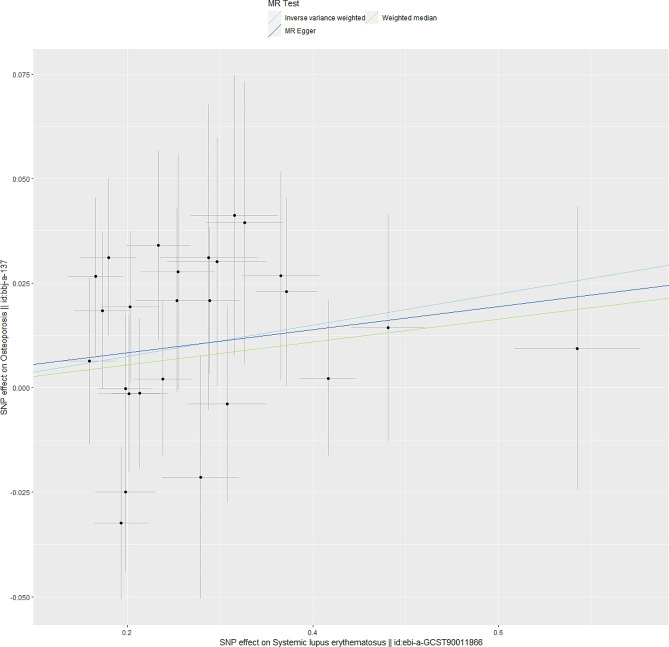
Scatter plots of the MR estimates for the association of SLE with osteoporosis. SLE, Systemic lupus erythematosus; MR, Mendelian randomization; SNPs, single nuclear polymorphisms

In the IVW analysis, there was evidence to support a significant association between SLE and risk of osteoporosis (OR = 1.038, SE = 0.016, *p* = 0.017). The weighted median approach provided no evidence of a significant causal association between SLE and osteoporosis (OR = 1.028, SE = 0.022, *p* = 0.213). The MR-Egger approach (OR = 1.028, SE = 0.044, *p* = 0.541), is aims to provide an estimate of the causal effect while allowing for possible pleiotropy, which is when genetic variants affect the outcome through multiple pathways, not only the one you are interested in. The results were shown in Table 1; Fig. 2. A *p*-value of > 0.05 from MR Egger suggests that the causal relationship is not significant after taking into consideration potential pleiotropic effects. In the subsequent sensitivity analysis, the MR Egger intercept (intercept = 0.003, SE = 0.012, *p* = 0.817), MR-PRESSO global test (*p* = 0.8) did not suggest evidence of horizontal or directional pleiotropy. The above analysis revealed that pleiotropy was unlikely to be biasing the results. Meanwhile, Cochran’s Q test for heterogeneity showed that there was no heterogeneity between IVs(Cochran’s Q = 19.34, *p* = 0.78) (Table 2).

**Table 1:** Causal efect between SLE and osteoporosis by diferent MR analysis methods

Exposure	Outcome	method	nSNP	OR	95% CI		SE	*p* value
SLE	Osteoporosis	Inverse variance weighted	26	1.038	1.007	1.070	0.016	0.017
SLE	Osteoporosis	Weighted median	26	1.028	0.984	1.073	0.022	0.213
SLE	Osteoporosis	MR Egger	26	1.028	0.942	1.122	0.044	0.541

SLE, Systemic lupus erythematosus; SNP, Single nucleotide polymorphism; SE, Standard Error; MR, Mendelian randomization

**Table 2:** Heterogeneity test of MR

Heterogeneity test	Q	df	Q_P Value
MR egger	19.29	24	0.737
IVW	19.34	25	0.780

MR, Mendelian randomization; IVW, inverse variance-weighted

**Fig. 3:**
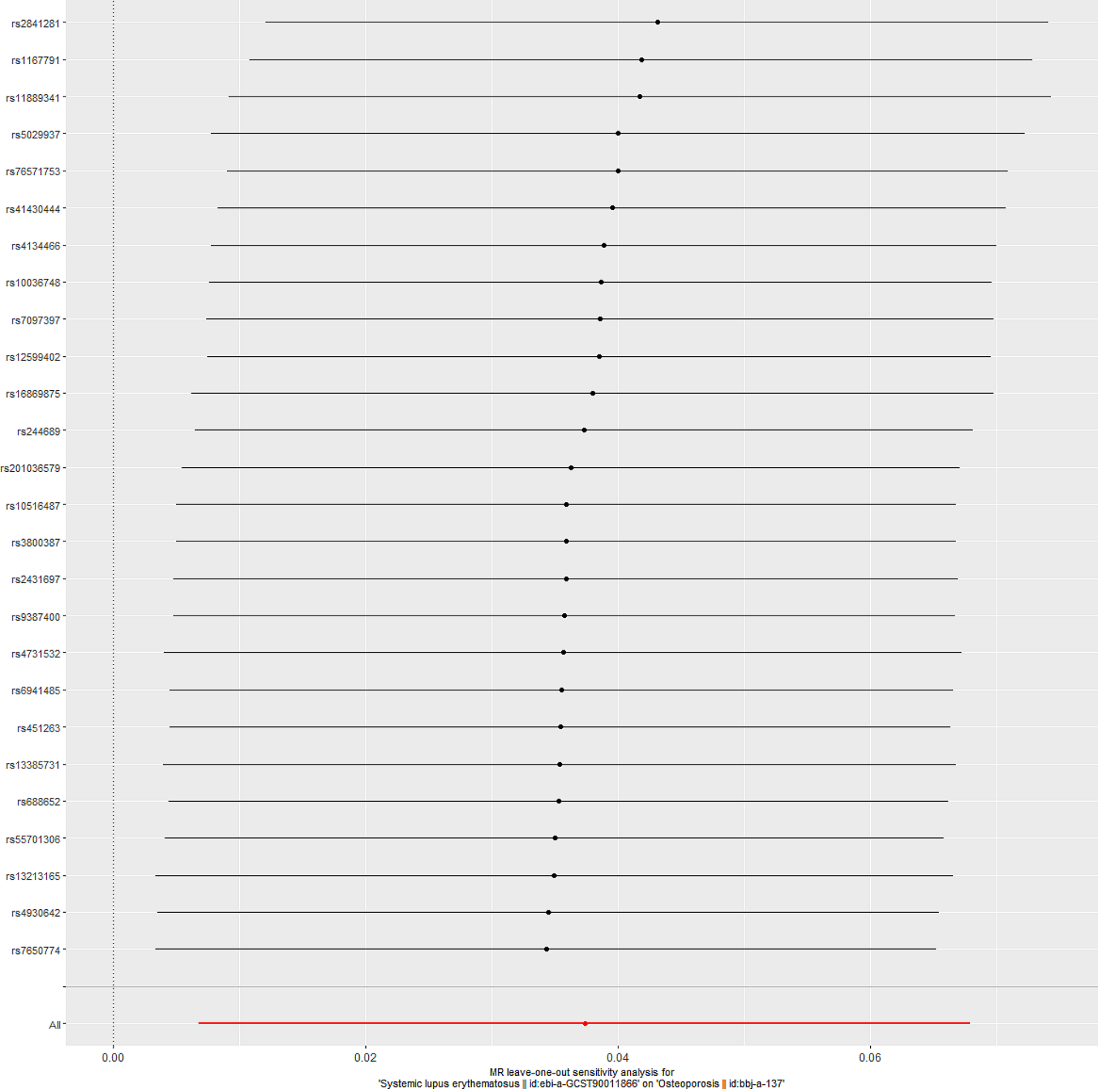
Leave-one-out plots for the MR analyses of SLE on osteoporosis. SLE, Systemic lupus erythematosus; MR, Mendelian randomization

**Fig. 4:**
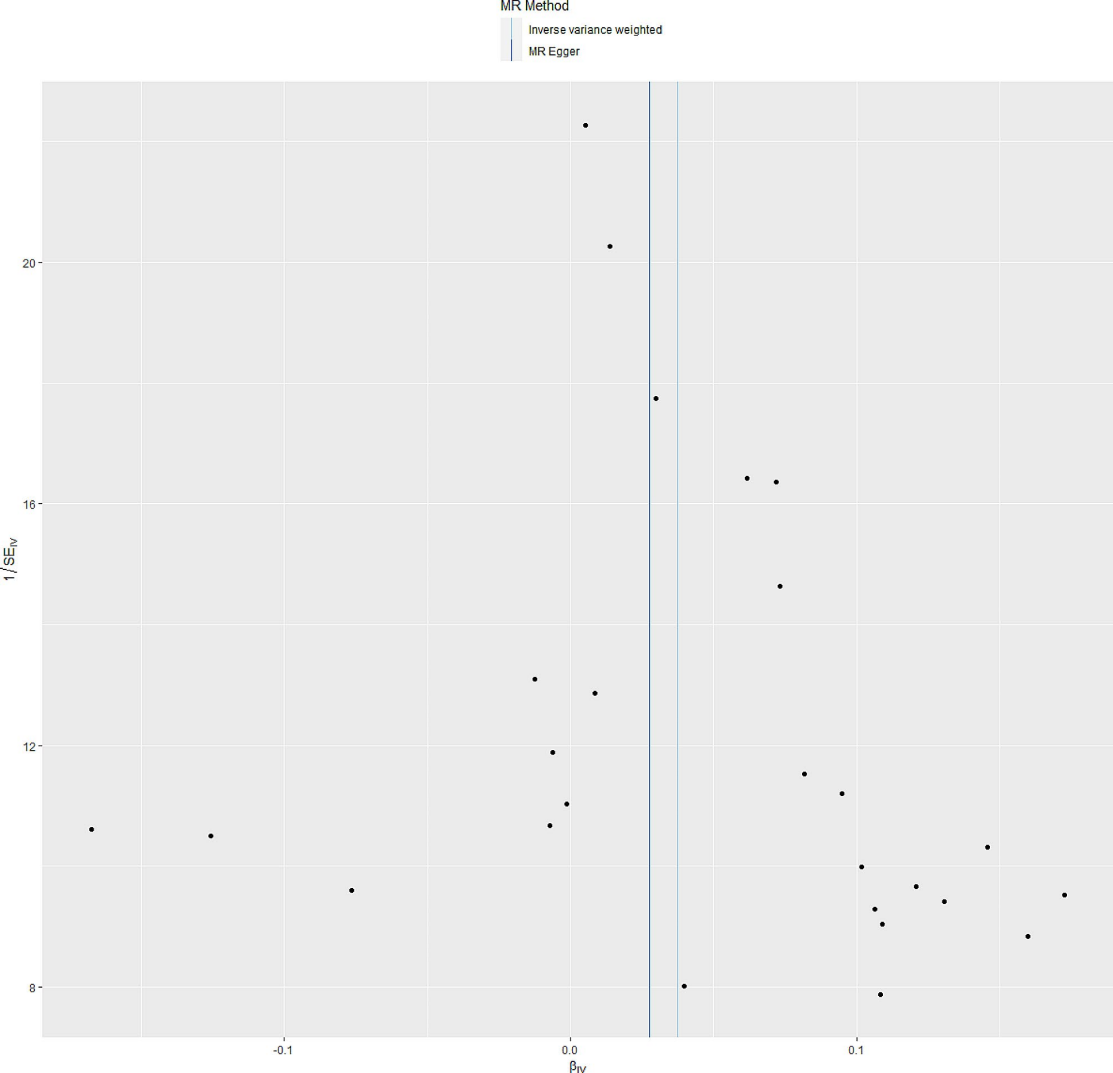
Funnel plot for the MR analyses of SLE on osteoporosis. SLE, Systemic lupus erythematosus; MR, Mendelian randomization

The “leave-one-out” sensitivity indicated that there was no single SNP to influence the causal effect between SLE and osteoporosis (Fig. 3). Furthermore, the funnel plot was symmetry, indicating no pleiotropy (Fig. 4).

Therefore, the MR analysis results support a potential association between SLE and osteoporosis.

## Discussion

To our knowledge, this is the first study that has used the MR approach to investigate the potential relationship between SLE and osteoporosis. Our results provide valuable insights into the association between these two complex diseases and may have significant implications for their prevention and treatment strategies.

First, it is essential to recognize that both SLE and osteoporosis are multifactorial diseases influenced by a multitude of genetic and environmental factors. Consequently, it is challenging to establish a clear causal relationship using conventional observational studies, which can be impacted by confounding and reverse causation. MR analysis offers a robust alternative by using genetic instruments as proxies for the exposure of interest, thereby minimizing biases and providing a more reliable assessment of causality [20].

The results of our MR analysis provide evidence for a potential relationship between SLE and an increased risk of osteoporosis. This supports the notion that the autoimmune-mediated inflammation characteristic of SLE may have a direct impact on bone metabolism and remodeling processes, potentially leading to the development of osteoporosis. Several possible biological mechanisms may underlie this potential relationship. One possible explanation for the link between SLE and osteoporosis is that chronic inflammation, a hallmark of SLE, can lead to bone loss [28]. Inflammation can activate bone-resorbing cytokines, which stimulate the activity of osteoclasts (cells that break down bone tissue) and inhibit the activity of osteoblasts (cells that build new bone tissue). This results in a net loss of bone mass over time, leading to a higher risk of osteoporosis. Several studies have reported that individuals with SLE have higher inflammatory cytokine levels than healthy controls, supporting this theory [28, 29]. Another potential mechanism is the use of corticosteroids in the management of SLE [30]. Corticosteroids are frequently used to control inflammation in SLE patients, but long-term use of these drugs increases the risk of osteoporosis [31]. Corticosteroids can interfere with the normal balance of bone remodeling, leading to decreased bone formation and increased bone resorption [32]. Moreover, renal involvement in SLE might contribute to secondary hyperparathyroidism, increased osteoclastic bone resorption, and reduced synthesis of 1,25(OH)2D, ultimately leading to a reduction in bone mass [33–37]. Lastly, limitations in physical activity and sunlight exposure among SLE patients could lead to reduced bone mineral density. These are all important mechanisms underlying the link between SLE and osteoporosis [38, 39]. However, more research is needed to elucidate the complex interactions among these factors and to develop more effective strategies for managing and preventing osteoporosis in SLE patients.

Given the potential association between SLE and osteoporosis identified here, it is imperative that clinicians and researchers be aware of this connection. Early identification of osteoporosis risk in SLE patients allows healthcare providers to monitor and implement preventive measures against the development of this condition [40, 41]. Preventative measures, such as promoting adequate calcium and vitamin D intake [42], encouraging regular weight-bearing exercise, and considering the use of bone-protecting medications, such as bisphosphonates and denosumab [43], may be crucial in mitigating the increased risk of osteoporosis in SLE patients [44].

Despite the strengths of MR analysis, some limitations should be acknowledged. First, potential pleiotropy, or the influence of genetic variants on multiple traits, may affect the validity of the association identified. The efects of SLE on osteoporosis are complicated and diverse, generally due to the combined action of various factors. Corticosteroids are often used to control inflammation and complications in SLE patients, which might affect bone mineral density in patients with SLE [45]. We could not eliminate the influence of confounding variables, which might lead to bias in the findings. Second, the extent to which our findings may be generalizable across diverse populations is unclear. As the genetic predisposition to SLE and osteoporosis may vary by race, ethnicity, and genetic background, replication in diverse cohorts is essential to determine the generalizability and robustness of our findings. Future MR studies incorporating more diverse populations are needed to validate these associations.

In conclusion, our MR analysis provides evidence supporting a potential relationship between SLE and osteoporosis. Our findings highlight the need for increased awareness regarding the potential risk of osteoporosis among SLE patients and recommend monitoring bone health as a part of routine SLE management. Further studies should refine genetic instruments, explore potential pleiotropic effects, and validate the findings in diverse populations.

### Electronic supplementary material

Below is the link to the electronic supplementary material.

Supplementary Material 1

## Data Availability

The GWAS summary datasets for SLE (GWAS ID: ebi-a-GCST9001186) and osteoporosis (GWAS ID: bbj-a-137) are available through the ieu open gwas project (https://gwas.mrcieu.ac.uk/datasets).
